# The associations of parental smoking, quitting and habitus with teenager e-cigarette, smoking, alcohol and other drug use in GUI Cohort ’98

**DOI:** 10.1038/s41598-023-47061-4

**Published:** 2023-11-16

**Authors:** Salome Sunday, Luke Clancy, Joan Hanafin

**Affiliations:** 1 TobaccoFree Research Institute Ireland, FOCAS Institute, Technological University Dublin (TU Dublin), Dublin, Ireland; 2 Department of Sociology, University of Limerick, Limerick, Ireland

**Keywords:** Environmental social sciences, Risk factors

## Abstract

We analyse parental smoking and cessation (quitting) associations with teenager e-cigarette, alcohol, tobacco smoking and other drug use, and explore parental smoking as a mechanism for social reproduction. We use data from Waves 1–3 of *Growing Up in Ireland* (Cohort ’98). Our analytic sample consisted of n = 6,039 participants reporting in all 3 Waves. Data were collected in Waves 1 and 2 when the children were 9 and 13 years old and in Wave 3 at age 17/18 years. Generalized Estimating Equations (GEE) models were used to analyse teenage substance use at Wave 3. Parental smoking was associated with significantly increased risk of all teenage substance use, adjusted odds ratios were aOR2.13 (ever e-cigarette use); aOR1.92 (ever alcohol use); aOR1.88 (current alcohol use); aOR1.90 (ever use of other drugs); aOR2.10 (ever-smoking); and aOR1.91 (current smoking). Primary caregiver smoking cessation (quitting) was associated with a lower risk for teenager current smoking aOR0.62, ever e-cigarette use aOR 0.65 and other drug use aOR 0.57. Primary caregiver smoking behaviour had greater associations than secondary, and age13 exposure more than age 9. Habitus seems to play a role and wealth was protective for teenage smoking. The findings suggest that prevention interventions should target both caregivers and their children.

## Introduction

The period of adolescence is a critical developmental phase often associated with a predisposition to risk-taking and unhealthy behaviours such as substance use^1^. Globally, substance use is regarded as a leading public health problem because of serious health associations, social consequences, and due to its prevalence especially among teenagers^2–4^.

The 2019 report of the European School Survey Project on Alcohol and Other Drugs (ESPAD), indicates high levels of substance use among 15–16 year old students in Europe^5^. According to the report, about 79% of students in the 35 ESPAD participating countries had tried alcohol in their lifetime, 40% had tried e-cigarettes, and 41% had smoked cigarettes. The 2019 Irish wave of the ESPAD survey indicates that 76% of Irish students have tried alcohol, 39% have tried e-cigarettes and 18% are current e-cigarette users, 32% have ever smoked cigarettes and 14% are current smokers, 19% have tried cannabis and at least 3% have tried other drugs including cocaine, inhalants and ecstasy^6^.

Teenager substance use is a matter of particular concern because behaviours that start during adolescence such as cigarette smoking often persist and results in intergenerational cycles of nicotine dependence, substance use-related diseases and increased mortality^7^. It puts teenagers at risk of short-term problems such as poor school performance and truancy^6^ as well as long-term problems including impaired neurodevelopment and substance use disorders^8^.

Understanding the underlying factors associated with substance use is critical in reducing its prevalence and addressing substance use-related problems among teenagers.

The impact of parental substance use on teenage substance use is one such factor^9–12^. As regards smoking, parental smoking appears to be a critical factor associated with intergenerational transmission of smoking^13^ and the association between parental smoking and teenage smoking has been frequently reported^9,12–15^. Gilman et al.^15^ describe the evidence of a relation between parental smoking and the risk of smoking initiation during adolescence as “compelling, although inconsistent” but Waa and colleagues found that parental smoking was not associated with teenager smoking^16^. On the other hand little is recorded about associations between parental smoking cessation (quitting) and teenage substance use including smoking and e-cigarettes.

As regards other teenage substance use, associations with parent smoking status are more limited but there is some evidence for an association between parental smoking and teenager alcohol and other drug use^17^. Also, there is some emerging evidence from the UK^18^ and from Taiwan and West Malaysia pointing to links between parental smoking and teenager e-cigarette use^19–21^. This is of particular interest in this study because, for the first time in 25 years, there has been an increase in teenage smoking in Ireland associated with a dramatic rise in e-cigarette use in Irish teenagers^22^. Some authors have theorised that the mechanisms underlying these observed associations are either as a result of a transfer of genes from smoking parents to their children, thereby increasing their liability to smoke and subsequently use other drugs^23^ and/or the imitation of particular personality characteristics of parents^24^. The exposure of children to secondhand smoke and therefore nicotine from parental smoking is a further possible factor in e-cigarette use and smoking^25^.

More recently, health interest has increased in the “social context of smoking”^26^ in addition to the physiological dimensions of addiction, as it is seen as key to understanding two vital public health phenomena, the growing concentration of smoking among socially and economically marginalised groups, and understanding and addressing diverse sources of resistance to tobacco control^26^. Differences in smoking and other “lifestyle behaviours” such as alcohol use play a role in continued health inequalities^27^. Mindful of how health (and other) inequalities persist intergenerationally, social reproduction theorists grapple with explanations of intergenerational inequalities. Concepts developed by Bourdieu to explain social and cultural reproduction, including habitus, field, practice, and capital, have recently been used to explain health-related practices including teenagers’ and young people’s smoking and alcohol use^28–31^. Because smoking and other substance use are major contributory factors to health inequalities, we suggest the potential usefulness of this theoretical framework in this context and have explored it in this study.

For our emphasis on parent smoking, the Bourdieusian concept of habitus is of particular use. Habitus refers to an individual’s dispositions, beliefs, values, and habits engrained or inculcated by life experience^32^. Bourdieu also delineates forms of capital beyond economic capital (social, cultural, symbolic) which interact with this “system of dispositions” called habitus. Habitus operates at a subconscious level, and structures perceptions and practices^33^, predisposing people to behave in particular ways^34^. Habitus is acquired in the home, and is further influenced by school, work, and all subsequent life experiences^35^. Thus, parents and caregivers are particularly important in teenagers’ enculturation into practices of substance use.

Using a large nationally representative cohort sample, this study examines four facets of parental smoking and teenage substance use. First, we examine whetherliving with a parent who smokes when a child is aged 9 or 13 years (childhood and early adolescence) increases the risk of subsequent teenage substance use (cigarettes, e-cigarettes, alcohol and other drug use) at age 17/18 years, and, if yes, whether this risk differs if one or both parents smoke when the child is aged 9 *or* 13 years, and whether the risk increases when each parent is reported smoking at 9 *and* 13 years. These circumstances have not previously been explored in the literature on the established links between parental smoking and teenage substance use. Secondly, we focus on associations between parental smoking and teenager e-cigarette use about which little is known. Thirdly, we examine whether having a parent who quits smoking reduces the likelihood of teenage smoking, e-cigarette and other drug use. Fourthly, in line with our interest in Bourdieusian social reproduction theory, we examine whether social class capitals (family affluence and parental education) are associated with teenage substance use independently of parental smoking. These data and analyses will provide more robust evidence about familial contexts to help explore these relationships, to inform subsequent research, and also to inform substance use cessation interventions both to help prevent initiation and encourage cessation of substance use in teenagers.

## Methods

### Data

The data are from the Growing up in Ireland National Longitudinal Study of Children (GUI), a nationally representative cohort study of children living in the Republic of Ireland. GUI follows two cohorts of children: an infant and child cohort. We use the Child Cohort (Cohort ’98) which commenced in 2008 when the children were aged 9 years (Wave 1). A two-stage clustered sampling method was used to recruit 8,568 children from the national primary school system born between November 1997 and October 1998 and their caregivers (Wave 1).

We use data from Wave 1 (2008, children aged 9 years), Wave 2 (aged 13 years) and Wave 3 (aged 17/18 years). The two-stage sampling approach generated a random sample of 910 primary schools (82% response rate) in Wave 1 comprising n = 8,568 9-year-old children and their families. The second study wave was carried out in 2011/2012 (Wave 2) when the children were aged 13 with an 89% follow-up rate (*n* = 7,525). The third study wave was carried out in 2016 when the children were aged 17/18 years (n = 6,216) representing a 81% follow-up rate.

The lower sample size in waves subsequent to Wave 1 can be attributed to several factors. These include respondents and their families declining to participate, respondents and their families no longer residing in Ireland during the waves, making them ineligible for inclusion in the target population. Additionally, some participants might have moved without providing a forwarding address, possibly relocating outside the country. Others could not be contacted or were unavailable during the data collection period. Furthermore, certain families had invalid addresses or explicitly requested not to be approached for further waves of the study^36,37^.

Parental smoking data was extracted from the Parent questionnaires in Waves 1 and 2. Demographic variables (gender, region, parental education, household income quintile, household type) were also extracted from Wave 1 Parent questionnaires. To examine the associations between social class and smoking we used measures of household affluence, income quintiles and parental education.

Teenage substance use was extracted from the Wave 3 Young Person Sensitive Questionnaire as were their reports of their peers smoking. Peer smoking data were not collected in Waves 1 or 2. Data collection for children and their parents/caregivers was performed at each family’s residence by trained interviewers.

A full description of the study including the design, instruments and data collection procedures have been described elsewhere^36–38^. The data, in the form of a Researcher Microdata File (RMF), are archived in the Central Statistics Office and are available to researchers on request and following a formal application, training and approval process.

### Representativeness of the analytic sample

Our analytic sample comprises 6039 participants in Wave 3 who were also present in Waves 1 and 2, and represent 70% of the original sample in Wave 1. The teenagers and their parents in the analytic sample were comparable to the baseline cohort based on the distribution of background characteristics, such as household income, gender, and parents’ level of education. This indicates that the analytic sample is representative of the baseline sample, allowing for meaningful comparisons to be made in our study. We show this in Table 1 below (which describes the sample characteristics).

**Table 1:** Characteristics of the baseline and analytic samples.

Variable	Baseline sample (n = 8568)	Analytic sample (n = 6039)
Gender		
Male	4377 (51.1)	3072 (50.9)
Female	4190 (48.9)	2967 (49.1)
Primary caregiver level of education		
Junior certificate	2584 (30.2)	1760 (29.2)
Leaving certificate	3145 (36.7)	2239 (37.1)
Certificate/diploma	1363 (15.9)	993 (16.5)
Degree	1476 (17.2)	1045 (17.3)
Secondary caregiver level of education		
Junior certificate	2442 (34.3)	1733 (33.4)
Leaving certificate	2057 (28.9)	1524 (29.3)
Certificate/diploma	1078 (15.2)	783 (15.1)
Degree	1538 (21.6)	1156 (22.2)
Household income quintiles		
Lowest	1584 (19.9)	1020 (18.2)
Second	1594 (20.1)	1178 (21.0)
Third	1594 (20.1)	1147 (20.4)
Fourth	1580 (19.9)	1137 (20.3)
Highest	1588 (20.0)	1131 (20.2)
Household type		
One parent	1553 (18.1)	1047 (17.3)
Two parents	7014 (81.9)	4992 (82.7)
Region		
Urban	3830 (44.8)	2648 (44.0)
Rural	4719 (55.2)	3373 (56.0)

### Study participants and data collection

The 6039 respondents who participated in all three waves of the GUI child cohort survey at 9 years (Wave 1, 2008), at 13 years (Wave 2, 2012) and at 17/18 years (Wave 3, 2016) were included in this study. At each wave, the study child and one or both parents (where there were two parents/caregivers) were asked to self-complete a paper-based main questionnaire and a sensitive questionnaire. In addition to the questionnaires, computer assisted personal interviews were conducted in the family`s home. The GUI study categorizes parents into primary and secondary caregivers. A primary caregiver was defined as the person who provided most care and who knew most about the Study Child, the mother or mother figure for 98% of our participants. The secondary caregiver was defined as the spouse or partner of the primary caregiver, usually the child’s father or father figure^37^. In the baseline sample 82% (n = 7014) of children, and in the analytic sample 83% (n = 4992) of children were in households with a secondary caregiver (i.e., with two caregivers). Ethical approval for the GUI project was granted by the Irish Health Research Board`s Research Ethics Committee and we confirm that all methods were performed in accordance with the relevant guidelines and regulations. Informed consent was obtained from all subjects and/or their legal guardian(s) (e.g., https://www.growingup.gov.ie/pubs/9_Year_CC_Parent_Leaflet.pdf).

## Measures

### Exposure variable: caregiver smoking

At Waves 1 and 2, caregivers were asked separately to report if they currently smoke daily, occasionally, or not at all. Their responses were recoded into: Yes (daily/occasionally) and No (not at all).

### Outcome variables: substance use at age 17/18 (Wave 3)

Substance use was reported by the teenagers in Wave 3 using the following questions.

#### Smoking

##### Ever-smoking

“Have you ever smoked a cigarette?”, response options: “Yes” and “No”.

##### Current smoking

Those who responded yes to ever smoking a cigarette were asked: “Which of the following best describes you?”, response options: “Only ever tried smoking once or twice”, “Used to smoke but not now”, “Smoke occasionally”, “Smoke daily”, “Don’t smoke”. Respondents indicating that they smoked occasionally or daily were classified as “current smokers” while all other responses were classified as “non-current smokers”.

#### E-cigarette use

##### Ever e-cigarette use

“Have you ever tried an e-cigarette or vaping”, response options “Yes” and “No”.

*E-cigarette current use* was not measured in the GUI survey.

#### Alcohol consumption

##### Ever alcohol use

“Have you ever consumed alcohol”, the response options were: “Yes” and “No”.

##### Alcohol current use

Those who responded yes to ever alcohol use were asked: “How often have you consumed alcohol”. Response options: “Never”, “Monthly or less”, “2–4 times a month”, “2–3 times a week”, “4+ times a week”. Respondents indicating that they had consumed alcohol Monthly or less, 2–4 times a month, 2–3 times a week, 4+ times, were recoded as current alcohol users.

#### Use of other drugs

##### Ever-use of other drugs

**“**Have you ever tried cannabis (also called marijuana, hash, dope, pot, skunk, puff, grass, draw, ganja, spliff joints, smoke, weed)?” “Have you ever tried inhaling or sniffing aerosols/ gas (lighter refills)/ glue/solvents?” “Have you ever tried, taken or used any non-prescribed drugs, such as ecstasy, cocaine, heroin, etc?”

Respondents indicating that they had tried any of these drugs were classified as other drug ever-users.

*Current use of other drugs* was not measured in the GUI survey.

#### Demographic characteristics

The following variables, assessed at Wave 1 in the parent questionnaire, were included as covariates for teenage substance use, based on results from previous studies^39,40^:

Teenager Gender (M/F), Income (equivalized to account for household size and composition using the modified Organisation for Economic Co-operation and Development equivalence scale and presented in quintiles), Region (urban/rural), Household type (one parent, one child; one parent, two or more children; two parents, one child; two parents, two or more children, recoded into one-parent families, two-parent families), primary and secondary parents’ educational level (primary or less; intermediate/ junior/ group certificate or equivalent, leaving certificate or equivalent, diploma/certificate, primary degree, postgraduate/higher degree, recoded into primary and lower secondary, upper secondary, further education and higher education). The variable “household type” was excluded from the GEE models due to its collinearity with other variables.

### Peer smoking

Peer smoking was only recorded by respondents in the Young Person Sensitive questionnaire in Wave 3 (17/18-year-olds). Teenagers were asked to report how many of their regular friends do or have ever smoked cigarettes. The response options were: “None”, “A few”, “Some”, “Most”, “All”, recoded into None/A few vs Some/Most/All.

### Parental smoking cessation (quitting)

This sample consisted of (n = 2481) caregivers who smoked at Wave 1, (n = 1445) primary caregivers and (n = 1036) secondary caregivers and was drawn from those respondents where at least one caregiver reported smoking at baseline. Both parents were smokers in 345 households.

### Statistical analysis

We first applied Pearson’s chi-square tests to test for the associations between parental smoking at Waves 1 and 2 and teenage substance use at 17/18 years, allowing us to compare departures from the expected distributions. This was followed by a series of GEE models to compare longitudinally the relationship between parental smoking and teenage substance use.

Firstly, exposure to caregivers who smoked was dichotomized (yes vs no) for each of the three caregiver smoking categories (*primary caregiver smoked*, *secondary caregiver smoked*, and *both caregivers smoked*) at Wave 1 and 2 and repeated for each teenage substance use variable at Wave 3 (*teenager ever-smoking*, *current smoking*, *ever e-cigarette use*, *ever alcohol use*, *current alcohol use*, and *ever use of other drugs*).

To account for exposure to smoking at both childhood (Wave 1) and early adolescence (Wave 2), a variable was created for caregivers (primary, secondary, both) who *smoked at both waves*. This was defined based on the responses (yes vs no) to primary, secondary and both caregivers reporting being a smoker at both Waves 1 and 2 against teenager substance use at Wave 3.

Finally, a new exposure variable was created to assess associations between teenage substance use and having a primary *caregiver quit, a* secondary caregiver quit or *both caregivers quit,* smoking. This was defined based on the responses (yes vs no) to, primary, secondary or both caregivers smoking at Wave 1 but not smoking at Wave 2.

The covariates identified as gender (male vs females), parental education (none/primary/secondary/upper level), income (in quintiles), peer smoking (none/at least one vs some/most/all) and region (urban vs rural) were included in the model.

All associations are reported as adjusted odds ratios (aOR) and 95% confidence intervals (95% CI), and a P < 0.05 was considered statistically significant. Weighting, a minimum information loss algorithm developed by GUI, was used to adjust for differences between the GUI sample and the population at age 9, 13 and 17/18 years. These weights were applied in all analyses to enable inferences to be made about the entire population from which the study participants were selected. Further details regarding weighting in GUI can be found here: www.gui.com/guide-to-datasets/. All analyses were performed using STATA version 16.1.

## Results

### Characteristics of study participants

The characteristics of the teenagers and their caregivers in the baseline (wave 1) and analytic samples (all responding at wave 3) are presented in Table 1 below.

**Figure 1:**
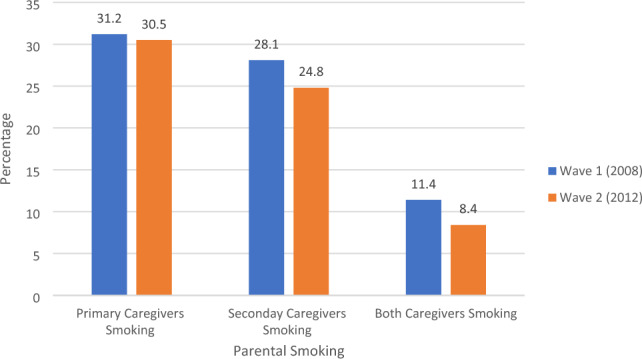
Prevalence of parental smoking in wave 1, and 2 (analytic sample, n = 6039).

Figure 1 presents the prevalence of caregiver smoking in the analytic sample (n = 6,039) in Wave 1 and Wave 2. Declining smoking rates in the adult population over the period were evident in both primary and secondary caregivers. Among primary caregivers, smoking prevalence was highest in Wave 1 (31.2%, n = 1883), and was 30.5% (n = 1829) in Wave 2. Among secondary caregivers, 28.1% (n = 1458) reported smoking in Wave 1, and 24.8% (n = 1143) in Wave 2. In wave 1, 11.4% (n = 688) of both caregivers were smokers, and this figure decreased to 8.4% (n = 506) in Wave 2.

### Bivariate associations between parental smoking and teenage substance use

Table 2 presents the bivariate associations between parental smoking and teenager substance use at 17/18 years which indicate significantly higher substance use for all substances examined among teenagers whose caregivers had smoked when the teenagers were 9 years old (Wave 1) or when they were 13 years old (Wave 2). Alcohol was the most used substance. One in three reported ever e-cigarette use while half had ever-smoked and one in five were current smokers More than a third reported ever use of other drugs.

**Table 2:** Bivariate associations of teenager substance use at 17/18 years and parental smoking.

Teenager substance use	Total N (%) Substance use at age 17/18 years	Primary caregiver smoking Wave 1 N (%), n = 1883, 31.2%	Primary caregiver smoking Wave 2 N (%), n = 1829, 30.5%	Secondary caregiver smoking Wave 1 N (%), n = 1458, 28.1%	Secondary caregiver smoking Wave 2 N (%), n = 1143, 24.8%	Both caregivers smoking Wave 1 N (%), n = 688, 11.4%	Both caregivers smoking Wave 2 N (%), n = 506, 8.4%
Ever-smoking							
No	3026 (50.7)	**804 (26.6)**	**797 (26.6)**	**647 (24.3)**	**493 (20.5)**	**276 (9.1)**	**197 (6.5)**
Yes	2948 (49.3)	**1051 (35.6)**	**1006 (34.3)**	**798 (32.1)**	**640 (26.7)**	**405 (13.7)**	**307 (10.4)**
Current smoking							
No	4760 (79.7)	**1355 (28.5)**	**1310 (27.8)**	**1093 (26.2)**	**832 (22.3)**	**490 (10.3)**	**351 (7.4)**
Yes	1213 (20.3)	**498 (41.1)**	**494 (40.8)**	**351 (36.2)**	**302 (36.6)**	**192 (15.8)**	**152 (12.5)**
Ever e-cigarette use							
No	3954 (66.1)	**1114 (28.2)**	**1080 (27.5)**	**853 (24.7)**	**693 (22.4)**	**364 (9.2)**	**279 (7.0)**
Yes	2024 (33.9)	**742 (36.6)**	**725 (36.0)**	**593 (35.2)**	**441 (30.1)**	**318 (15.7)**	**226 (11.1)**
Ever alcohol use							
No	627 (10.5)	**141 (22.5)**	**157 (26.0)**	**143 (25.8)**	**91 (18.9)**	**47 (7.5)**	**34 (5.4)**
Yes	5351 (89.5)	**1714 (32.0)**	**1647 (30.9)**	**1303 (28.4)**	**1043 (25.6)**	**635 (11.9)**	**470 (8.8)**
Current alcohol use							
No	910 (15.2)	**224 (24.6)**	**239 (27.0)**	**190 (24.7)**	**129 (19.1)**	**73 (8.1)**	**50 (5.5)**
Yes	5065 (84.8)	**1631 (32.2)**	**1564 (31.0)**	**1255 (28.7)**	**1005 (25.9)**	**609 (12.0)**	**455 (9.0)**
Ever use of other drugs							
No	3941 (65.3)	**1087 (27.6)**	**1075 (27.5)**	**890 (25.4)**	**689 (22.1)**	**402 (10.2)**	**291 (7.4)**
Yes	2098 (34.7)	**796 (38.0)**	**754 (36.2)**	**568 (33.4)**	**454 (30.6)**	**286 (13.7)**	**215 (10.2)**

Figures in bold are statistically significant at p < 0.05.

Table 3 below shows that, after adjusting for the covariates, smoking in primary, secondary and both caregivers at Waves 1 and Wave 2 was associated with higher adjusted odds ratios (aORs) of teenager ever e-cigarette use ranging from aOR1.33 to aOR1.88. Primary and both caregivers smoking at waves 1 or 2 was also associated with significantly higher aORs of all other variables tested with aORs ranging from aOR1.36 to aOR1.99. Secondary caregiver smoking in Wave 1 was not significantly associated with ever or current alcohol use nor with use of other drugs in Wave 2 but was associated with all other variables.

**Table 3:** Adjusted odds ratios (aOR) with 95% confidence intervals (CI) of caregiver smoking at Wave 1 and Wave 2 and teenager substance use at Wave 3 (17/18) years

Caregiver smoking status at Wave 1 (child aged 9 years)				Caregiver smoking status at Wave 2 (child aged 13 years)		
Teenager substance use	Primary caregiver smoking (n = 1883, 31.2%)	Secondary caregiver smoking (n = 1458, 28.1%)	Both caregivers smoking (n = 688, 11.4%)	Primary caregiver smoking (n = 1829, 30.5%)	Secondary caregiver smoking (n = 1143, 24.8%)	Both caregivers smoking (n = 506, 8.4%)
	aOR (95% CI)	aOR (95% CI)	aOR (95% CI)	aOR (95% CI)	aOR (95% CI)	aOR (95% CI)
Ever-smoking						
Yes	**1.60 (1.30, 1.98)**	**1.35 (1.17, 1.57)**	**1.56 (1.29, 1.88)**	**1.65 (1.42, 1.93)**	**1.55 (1.30, 1.84)**	**1.93 (1.53, 2.44)**
No	Reference	Reference	Reference	Reference	Reference	Reference
Current smoking						
Yes	**1.78 (1.49, 2.13)**	**1.42 (1.12, 1.81)**	**1.69 (1.25, 2.29)**	**1.99 (1.55, 2.56)**	**1.79 (1.36, 2.35)**	**1.88 (1.32, 2.69)**
No	Reference	Reference	Reference	Reference	Reference	Reference
Ever e-cigarette use						
Yes	**1.68 (1.44, 1.96)**	**1.60 (1.38, 1.86)**	**1.88 (1.56, 2.28)**	**1.76 (1.50, 2.06)**	**1.33 (1.00, 1.76)**	**1.72 (1.37, 2.15)**
No	Reference	Reference	Reference	Reference	Reference	Reference
Ever alcohol use						
Yes	**1.59 (1.23, 2.06)**	1.01 (0.81, 1.28)	**1.52 (1.09, 2.11)**	**1.32 (1.02, 1.70)**	**1.39 (1.07, 1.81)**	**1.60 (1.06, 2.41)**
No	Reference	Reference	Reference	Reference	Reference	Reference
Current alcohol use						
Yes	**1.61 (1.29, 2.00)**	1.10 (0.90, 1.34)	**1.36 (1.03, 1.79)**	**1.36 (1.09, 1.69)**	**1.31 (1.03, 1.67)**	**1.42 (1.01, 1.99)**
No	Reference	Reference	Reference	Reference	Reference	Reference
Ever use of other drugs						
Yes	**1.76 (1.36, 2.29)**	**1.52 (1.18, 1.96)**	**1.52 (1.12, 2.06)**	**1.70 (1.31, 2.21)**	1.13 (0.83, 1.53)	**1.44 (1.01, 2.05)**
No	Reference	Reference	Reference	Reference	Reference	Reference

Adjusted for gender, caregivers’ education, peer smoking (Wave 3), household income, and region.

Adjusted odds ratios (aOR) in bold are significantly different to those of the reference category (P < 0.05).

For primary and secondary caregivers’ smoking the strongest associations were with teenager current smoking and e-cigarette use and other drugs.

In general there was a stronger association with primary caregiver smoking and teenagers’ substance use than with secondary caregiver smoking, and also stronger associations with Wave 2 rather than Wave 1 for each caregiver.

### Parental smoking cessation (quitting) and teenager substance use

We examined associations between having a caregiver or caregivers who had quit smoking by Wave 2 and teenage e-cigarette use, current smoking and use of other drugs at Wave 3 aged 17/18. There were 1445 primary caregivers and 1036 secondary caregivers in the analytic sample who smoked at Wave1 (2481 in total) and 345 of them were in households were both parents were smokers.

Table 4 shows that, in this sub-sample, there was a reduction in the number of teenagers who had caregivers smoking in Wave 2 compared with Wave (n = 271 primary; n = 304 secondary caregivers). As regards teenagers both of whose parents smoked, there were n = 42 fewer of these in Wave 2 than there were in Wave 1.

**Table 4:** Parental smoking cessation and teenager substance use at 17/18 years.

Teenage Substance use	Primary caregiver quit smoking in Wave 2 (n = 271, 18.8%)	Secondary caregiver quit smoking in Wave 2 (n = 304, 29.4%)	Both caregivers quit smoking in Wave 2 (n = 42, 12.4%)
	aOR* (95% CI)	aOR* (95% CI)	aOR* (95% CI)
Current smoking			
Yes	**0.62 (0.42, 0.92)**^**#**^	**0.60 (0.41,0. 88)**	**0.19 (0.05, 0.60)**
No	**Reference**	**Reference**	**Reference**
Ever e-cigarette use			
Yes	**0.65 (0.47, 0.90)**	1.00 (0.74, 1.38)	0.72 (0.36, 1.45)
No	**Reference**	Reference	Reference
Ever use of other drugs			
Yes	**0.57 (0.33, 0.99)**	1.09 (0.64, 1.84)	0.41 (0.09, 1.79)
No	**Reference**	Reference	Reference

*Adjusted for gender, caregivers’ education, peer smoking (Wave 3), household income, and region.

^#^Adjusted odds ratios (aOR) in bold are significantly different to those of the reference category (P < 0.05).

Primary, secondary and both caregivers quitting was significantly associated with reduced risk for teenage current smoking aOR: 0.62, 0.60, and 0.19 respectively. Ever-e-cigarette use aOR: 0.65, and ever-use of other drugs aOR: 0.57 were also significantly associated with primary caregiver quitting. Secondary or both caregivers quitting did not show a significant association in e-cigarette or other drug use in teenagers.

### Social class, parental smoking and teenager e-cigarette use and smoking

Chi-square tests (Table 5) show that wealthier caregivers are significantly less likely to smoke than are poorer caregivers. For teenagers, Table 6 shows that no statistically significant social class differences emerged for teenage ever- e-cigarette use, ever-smoking or current smoking. As regards current smokers, however, there was a small but not statistically significant finding for teenagers in the poorest families to be current smokers and teenagers in the wealthiest families not to be (Table 6). These figures may suggest that although better-off teenagers are more likely to try smoking, possibly because of access, they are less likely to become smokers possibly because of lower caregiver smoking. Although these figures may be indicative of the very high numbers of teenagers who try smoking but do not continue smoking, they may also suggest an early manifestation of social reproduction.

**Table 5:** Associations between parental smoking status and household income at Wave 1.

	Wave 1 Household income quintile Lowest N (%)	Wave 1 Household income quintile 2nd N (%)	Wave 1 Household income quintile 3rd N (%)	Wave 1 Household income quintile 4th N (%)	Wave 1 Household income quintile Highest N (%)	Total N (%)
Primary caregiver smoking at Wave 1						
No	566 (14.7)	725 (18.9)	793 (20.6)	828 (21.6)	928 (24.2)	**3841 (68.5)**
Yes	454 (25.6)	452 (25.5)	354 (20.0)	308 (17.4)	203 (11.5)	**1771 (31.5)**
Secondary caregiver smoking at Wave 1						
No	**393 (11.3)**	634 (18.2)	719 (20.7)	835 (24.0)	**893 (25.7)**	**3474 (71.8)**
Yes	**256 (18.8)**	316 (23.2)	301 (22.1)	270 (19.8)	**221 (16.2)**	**1364 (28.2)**
Both caregiver smoking at Wave 1						
No	881 (17.7)	1019 (20.5)	997 (20.1)	1011 (20.4)	1057 (21.3)	4965 (88.5)
Yes	139 (21.5)	158 (24.4)	150 (23.2)	125 (19.4)	75 (11.5)	648 (11.5)

Significant values are in bold at p < 0.05.

**Table 6:** Associations between teenager substance use at Wave 3 and parent income (Wave 1).

	Wave 1 Household income quintile Lowest N (%)	Wave 1 Household income quintile 2nd N (%)	Wave 1 Household income quintile 3rd N (%)	Wave 1 Household income quintile 4th N (%)	Wave 1 Household income quintile Highest N (%)	Total N (%)
Teenage ever e-cigarette use						
No	689 (18.8)	766 (20.9)	738 (20.1)	736 (20.1)	736 (20.1)	3665 (66.0)
Yes	309 (16.3)	398 (21.1)	394 (20.8)	398 (21.0)	393 (20.7)	1892 (34.0)
Teenage ever smoking						
No	519 (18.6)	572 (20.5)	558 (20.0)	571 (20.5)	568 (20.4)	2788 (50.2)
Yes	478 (17.3)	593 (21.4)	573 (20.7)	562 (20.3)	559 (20.2)	2765 (47.8)
Teenage current smoking						
No	762 (17.3)	929 (21.1)	889 (20.1)	901 (20.4)	931 (21.1)	4412 (79.5)
Yes	236 (20.7)	235 (20.6)	242 (21.2)	231 (20.3)	196 (17.2)	1140 (20.5)

Results from the adjusted GEE analysis (not shown) confirm that being in a wealthier family was significantly associated with increased odds for teenage e-cigarette use with aORs in the highest household income quintile ranging from 1.53 to 1.58, compared to aORs 1.29 to 1.32 in the lowest income quintile, ever smoking aORs in the highest income quintile ranging from 1.28 to 1.47, ever alcohol use aOR ranging from 2.59 to 3.14 and current alcohol use aOR ranging from 2.31 to 2.79. Conversely, a protective effect of wealth for current smoking was also observed in this analysis, such that although teenagers whose caregivers smoked had higher odds of being current smokers, the risk was lower for teenagers in wealthier families with aORs ranging from 0.69 to 0.74.

Higher secondary caregiver education levels were significantly linked to lower odds of ever smoking (aOR0.69 to 0.73) and was associatedwith lower odds of teenage current smoking, with aORs ranging from 0.68 to 0.71 across all exposure categories. Lower educational levels were associated with lower odds of teenage e-cigarette use, alcohol consumption, and other drug use across all exposure categories.

In relation to region, results from our adjusted models indicate a protective effect of living in rural areas such that teenagers whose caregivers smoked were more likely to use e-cigarettes but those in rural areas were significantly less likely than those in urban areas (aORs ranging from 0.87 to 0.89). A similar finding was evident as regards the use of other drugs.

### Peer smoking

Associations with peer smoking was also examined in our adjusted analysis. In all analyses that we carried out, teenagers whose peers smoked (some/most/all) consistently had significantly higher odds of substance use in all adjusted models. As regards other covariate, however, associations were not homogenous across gender, social class and region. For example, after adjusting for other factors in the model, girls were significantly less likely to use e-cigarettes if their caregivers smoked or quit smoking than were boys.

## Discussion

The main aim of this study was to examine whether children’s exposure to caregivers smoking at ages 9 and 13 was associated with teenage (at age 17/18 years) e-cigarette, alcohol, and other drug use as well as smoking. While previous studies have documented the association between parental substance use and teenagers’ substance use^9,41,42^, this study examines exposures from the primary and secondary caregiver separately and also the effects of parental smoking cessation (quitting). Previous studies have either assessed smoking in one caregiver only^43^ or have not looked at smoking in caregivers separately, leading to an incomplete assessment of exposure to parental smoking. The longitudinal nature of our study also enabled us to investigate more nuanced effects of parental smoking on teenager substance use, taking account of both caregivers' smoking status over two waves of data collection four years apart and subsequent teenager substance use at a third wave four years later again. We found that teenagers whose caregivers smoked reported significantly higher odds of substance use (all six selected measures) compared with teenagers whose caregivers did not smoke or who stopped smoking.

As regards e-cigarettes we showed that exposure to either caregiver smoking at either wave (aged either 9 or 13 years) increases the risk of teenage ever e-cigarette use. Primary caregiver quitting was associated with significantly reduced odds of teenage e-cigarette use but secondary caregiver quitting was not. These novel findings highlight the role of parental smoking cessation as a potential intervention mechanism for preventing teenager e-cigarette use^40^.

We also found that exposure to parental smoking at either Wave is associated with teenager alcohol and other drug use at later adolescence, confirming the findings of Ferreira et al.^44^. We further report that greater exposure to parental smoking (e.g., both caregivers at both waves) is significantly associated with an increased risk of ever-use of alcohol and other drugs. Only alcohol current use was not significantly influenced by increased exposure to parental smoking and we enter a caveat and study limitation regarding the outcome variable *teenage alcohol use*, namely the absence of a variable on other parental substance use including alcohol itself. As parental smoking possibly co-varies with other parental substance use including alcohol and as parental alcohol may also co-vary with teenage alcohol use, the association with parental smoking may remain for teenage alcohol use. As our emphasis was on parental smoking associations in this study, we recommend a similar emphasis on parental alcohol use in future studies.

We confirm the results of some previous studies that parental smoking is associated with a significantly higher risk of smoking initiation in teenage offspring^13,41,45^ and agree with Gilman et al.^15^ that parental smoking “is an important source of vulnerability to smoking initiation among adolescents”. We also confirm findings of previous studies showing that the number of smoking parents and the persistence of parental smoking in both waves increases the likelihood of offspring smoking initiation^13,15,46^. Overall, therefore, we show that increased exposure to parental smoking is significantly associated with increased likelihood of teenager substance use. Others have suggested that this may be explained by how teenagers’ perceptions of parental norms influence their substance use. For example, Mehanovic and colleagues^47^ found that parental permissive norms toward cigarette smoking at baseline predicted adolescents’ illicit drug use at follow-up.

At a theoretical level, several aspects of the findings of this study provide support for the importance of the role of the family in shaping a young person’s habitus—their practice, dispositions, values and habits^32^ about tobacco use, e-cigarette use, alcohol and other drug use. First, the separate importance of both caregivers (and particularly the primary caregiver) in the increased odds of teenager substance use is noted. Secondly, the further increased odds of teenager substance use when both caregivers smoke, points to an aggregate effect on habitus within the familial field. Thirdly, there is an increased association over time with further increased odds of teenage substance use in households where caregivers smoked in both waves, pointing to a greater association with a more strongly developed habitus but this could also be an effect of more prolonged exposure to secondhand smoke^25^. Notably, our findings on the differentiated social class advantages (or disadvantages) that accrue to children from parental smoking cessation support theories of family habitus and social reproduction. For teenager current smoking, this class advantage was also evident even for those whose caregivers did not quit, being associated with both economic (wealth) capital and cultural (education) capital.

Overall, our findings suggest that teenage smoking is influenced by the combined effects of their experiences at home and with peers and support our assumption that parental smoking contributes significantly to patterns of smoking that are reproduced from one generation to the next. Our findings confirm lower smoking prevalence among wealthier parents and also indicate that parental quitting benefits children in wealthier families more than children in poorer families. These differential social class associations between parental quitting and teenage smoking and e-cigarette use lend further weight to how these classed reproduction processes help to explain the reproduction of social inequalities.

Thus, these findings add to what some have argued is the underestimated role of families in the reproduction of inequalities^48^ while also lending support to the growing body of work using Bourdieusian conceptual tools to explore how patterns of power and inequality are reproduced through the practices that are embedded in everyday life^49^. This may be relevant in health-related practices including teenagers’ and young people’s smoking^28,29^, young people’s alcohol use^31^, and teenage girls’ alcohol use^30^, a body of work that forms part of the shift within public health research from a biomedical model of illness and disease towards an understanding of the broader social determinants of health^26^.

Future tobacco prevention and cessation programs should not only be targeted at teenagers but also at their parents/caregivers. There is clear merit in interventions to support parents to stop smoking, even when the child has already been exposed because we found significantly reduced odds for teenage (17/18-year-old) e-cigarette use and current smoking when their parent(s) who had smoked when they were aged 9 years had quit by the time they were 13 years.

### Strengths and limitations

The main strength of this study is the use of data from a large nationally representative longitudinal study of children to examine the association of exposure to parental smoking with six teenager substance use outcomes. Previous studies have largely relied on cross-sectional data to examine this association^17,41^.

Parental smoking status was reported prospectively and separately by the caregivers, and teenage substance use was reported by the teenagers themselves as opposed to proxy reports of substance use. Research has shown that teenagers are not able to accurately report the extent of their caregivers’ substance use^41,50^. Secondly, this study controlled for the well-established confounding factors in the exposure-outcome relationship.

Our research also adds to a small number of previous papers using Bourdieusian theory to explore the role of smoking in the social reproduction of inequalities. Further research is also needed to examine the prospective associations between parental smoking and teenager substance use including the use of illicit drugs such as cannabis, as well as to develop and test theoretical models of the parents-teenagers smoking and other substance use link.

This study has several limitations which can be addressed in future research. First, there is a possibility of residual and/or unmeasured confounders. Studies such as this are unable to assess whether there are common determinants of parental and child substance use such as genetic liability. Secondly, teenagers’ substance use behaviours were based on self-report and were not validated by biochemical indicators such as cotinine measurement. However, previous studies have shown that self-reports are reliable and good indicators of substance use especially when carried out in total anonymity^51^. The GUI survey questions about substance use were asked using a sensitive questionnaire which the teenagers had to complete by themselves, and they were assured of confidentiality and anonymity. Additionally, caregivers and teenagers were both asked about the teenagers smoking habits and a moderate positive correlation was found between the teenager’s report of ever smoking and their caregiver’s report^36^.

### Implications

- We expand on previous research studies to demonstrate that exposure to either primary or secondary parental smoking, in either childhood and/or early adolescence, is linked with an increased risk of e-cigarette use in later adolescence, and of all substance-use measured. This should be considered for policy formation particularly in the rapidly changing field of e-cigarette use.
- We confirm for the first time in Ireland findings of previous studies linking parental smoking with teenage smoking. This has implications for policy and legislation in Ireland especially as regards the Endgame smoking policy of the Irish Government and for health education programmes in schools and in parent groups.
- Continuing exposure to parental smoking (each parent at each wave) increases the risk of all teenage substance use, with longer exposure(both waves) to parental smoking being significantly associated with an increased risk of smoking and ever-use of e-cigarettes, alcohol and other drugs.
- Parental quitting is positively linked with reduced teenage substance use, particularly for teenager current smoking and e-cigarette use.
- We use the Bourdieusian concept of habitus, a driver of teenager substance use, and report social class variations in the impact of parental smoking as well as the impact of parental quitting on teenager substance use. In general, children in wealthier families seem to benefit more from parental quitting than do children in poorer families. Such social class variations go some way towards explaining the intergenerational reproduction of inequalities.

## Data Availability

The data that support the findings of this study are available from Central Statistics Office Ireland but restrictions apply to the availability of these data, which were used under license for the current study, and so are not publicly available (https://www.cso.ie/en/aboutus/lgdp/csodatapolicies/dataforresearchers/rmfregister/).
